# Elevated Foxp3^+^ double-negative T cells are associated with disease progression during HIV infection

**DOI:** 10.3389/fimmu.2022.947647

**Published:** 2022-07-28

**Authors:** Leidan Zhang, Yuqing Wei, Di Wang, Juan Du, Xinyue Wang, Bei Li, Meiqing Jiang, Mengyuan Zhang, Na Chen, Meiju Deng, Chuan Song, Danying Chen, Liang Wu, Jiang Xiao, Hongyuan Liang, Hongxin Zhao, Yaxian Kong

**Affiliations:** ^1^ Peking University Ditan Teaching Hospital, Beijing, China; ^2^ Beijing Key Laboratory of Emerging Infectious Diseases, Institute of Infectious Diseases, Beijing Ditan Hospital, Capital Medical University, Beijing, China; ^3^ Beijing Institute of Infectious Diseases, Beijing, China; ^4^ National Center for Infectious Diseases, Beijing Ditan Hospital, Capital Medical University, Beijing, China; ^5^ Clinical and Research Center of Infectious Diseases, Beijing Ditan Hospital, Capital Medical University, Beijing, China

**Keywords:** HIV, Foxp3, double-negative T cell, immune activation, immune regulation

## Abstract

Persistent immune activation, which occurs during the whole course of HIV infection, plays a pivotal role in CD4^+^ T cells depletion and AIDS progression. Furthermore, immune activation is a key factor that leads to impaired immune reconstitution after long-term effective antiretroviral therapy (ART), and is even responsible for the increased risk of developing non-AIDS co-morbidities. Therefore, it’s imperative to identify an effective intervention targeting HIV-associated immune activation to improve disease management. Double negative T cells (DNT) were reported to provide immunosuppression during HIV infection, but the related mechanisms remained puzzled. Foxp3 endows Tregs with potent suppressive function to maintain immune homeostasis. However, whether DNT cells expressed Foxp3 and the accurate function of these cells urgently needed to be investigated. Here, we found that Foxp3^+^ DNT cells accumulated in untreated people living with HIV (PLWH) with CD4^+^ T cell count less than 200 cells/µl. Moreover, the frequency of Foxp3^+^ DNT cells was negatively correlated with CD4^+^ T cell count and CD4/CD8 ratio, and positively correlated with immune activation and systemic inflammation in PLWH. Of note, Foxp3^+^ DNT cells might exert suppressive regulation by increased expression of CD39, CD25, or vigorous proliferation (high levels of GITR and ki67) in ART-naive PLWH. Our study underlined the importance of Foxp3^+^ DNT cells in the HIV disease progression, and suggest that Foxp3^+^ DNT may be a potential target for clinical intervention for the control of immune activation during HIV infection.

## Introduction

Persistent immune activation, which occurs at the onset of HIV infection and persists in people living with HIV (PLWH) receiving effective antiretroviral therapy (ART) (1, 2), plays a key role in CD4 depletion and AIDS progression (3, 4). This condition may be associated with several factors including persistent antigen stimulation, microbial translocation, co-infections of other viruses, and cumulative ART toxicity (5). Indeed, immune activation is one of the strongest predictors of disease progression, is related to an increased risk of developing non-AIDS co-morbidities (6, 7), and is a key factor that leads to impaired immune reconstitution after long-term ART (8, 9). More importantly, the successful control of immune activation could be a target in the clinical therapy of PLWH.

Double negative T cells (DNT cells), which were acknowledged to express CD3 and TCRαβ, but not CD4, CD8 and CD56 surface markers (10, 11), constituted approximately 1-3% T lymphocytes in mice, as well as in humans, and an even higher percentage of T cells in some monkey species (12, 13). DNT cells derive either from the thymus by escaping negative selection or from peripheral T cells through downregulation of CD4 and CD8 and can be found in the peripheral blood, lymph nodes, and gut-associated lymphoid tissue (10, 11, 14–17). Previous studies identified the crucial role of DNT cells in graft-versus-host disease, autoimmune disease and infectious diseases, which were capable of limiting immune over-activation and maintaining immune system homeostasis (18–20). These cells exert immunosuppressive effects *via* various mechanisms, including high-level expression of CTLA-4 to downregulate the costimulatory molecules CD80 and CD86 on antigen-presenting mature DCs (21), production of anti-inflammatory cytokines such as IL-10, TGF-β (22), and induction of apoptosis *via* Fas/FasL pathway (23). Recently, accumulating evidence figured out a potential role of DNT cells to provide immunosuppression during HIV/SIV infection (15, 20, 22, 24), but the related mechanism needs to be further explored.

Foxp3, a 431 amino acid forkhead/winged-helix family transcriptional factor, predominantly expressed by a subset of CD4^+^ T cells, commonly referred as CD4^+^CD25^+^ regulatory T cells (25). It acts as a transcriptional repressor inhibiting the genes encoding synthesis of proinflammatory cytokines, while as an activator promoting the expression of canonical Treg cell components such as CD25, GITR, CD73, CD39 and CTLA-4, which endows the cell with powerful suppressive functions (26). Thus, Foxp3 plays a requisite role in humans for maintaining immunological self-tolerance state, thereby preventing the development of autoimmune diseases (27). Recent studies showed that the impact of Foxp3 on immune regulation was extended to non-CD4 immune cells such as CD8, γδT cells, and iNKT cells (28–30). Additionally, ectopic expression of Foxp3 mediated by retroviral gene transfer or cytokines induction in human non-Tregs was reported to yield a series of new cells with potent immune suppress function (31, 32). More importantly, several studies found a parallel elevation of Foxp3 expression in both CD4^+^ and CD8^+^ T cells from untreated PLWH as negative feedback of immune activation, which indicated a role of Foxp3^+^ T cells in HIV disease progression and the control of immune activation (33, 34). Nevertheless, there are limited data on the presence of Foxp3^+^ DNT cells in HIV infection. Vinton et al. found that there were a considerable number of Foxp3^+^ CD3^+^CD4^-^CD8^-^ T cells accumulating in most SIV-infected natural hosts with low peripheral immune activation (13). However, CD3^+^CD4^-^CD8^-^ T cells were acknowledged to be highly heterogeneous including not only DNT cells but also γδ T cells and iNKT cells (12), both of which definitely expressed Foxp3 (29, 30). Until now, previous studies have not provided sufficient evidence to support the expression of Foxp3 in DNT cells. Further investigations were urgently needed.

Here, we found that Foxp3^+^ DNT cells accumulated in untreated PLWH with low CD4^+^ T cells count in a persistent hyperinflammatory state and associated with disease progression. Furthermore, Foxp3^+^ DNT cells were also involved in the control of immune activation and inflammation during HIV infection. Finally, our data implied that these Foxp3^+^ DNT cells might exert suppressive regulation by elevated expression of CD39, CD25, or vigorous proliferation (high levels of GITR and ki67) as reported in Foxp3^+^ CD4^+^ Tregs in ART-naive PLWH.

## Materials and methods

### Study participants

We conducted a cross-sectional study, which was approved by the Committee of Ethics at Beijing Ditan Hospital, Capital Medical University in Beijing, and written informed consent was provided by each participant in accordance with the Declaration of Helsinki. In this study, we enrolled a cohort of 25 age-matched healthy controls (HCs), 121 treatment-naïve patients (TNs) and 32 HIV–infected patients receiving successful ART (ARTs). The precise infection time in these PLWH is unknown. At the time of specimen collection, ART-experienced PLWH were treated for a median of 5.2 years (IQR 4.1-6.4) with baseline CD4^+^ T cells count < 200 cells/µl and had plasma HIV RNA levels that were below detection according to routine clinical assays. TNs with CD4^+^ T cell count less than 200/µl are highly susceptible to opportunistic infections including tuberculosis and fungal infections. The clinical details of all the PLWH are shown in **Table 1**.

**Table 1:** Demographic and clinical characteristics of study participants.

Characteristics	HCs	TNs			ARTs	*P*-value*
		CD4 ≥ 350	200 ≤ CD4 < 350	CD4 < 200		
N (%)	25	42 (34.71)	30 (24.79)	49 (40.5)	32	–
Sex (M/F)	24/1	41/1	28/2	46/3	30/2	0.9252
Age (mean, years)	34 ± 7	29 ± 8	35 ± 8	40 ± 13	39 ± 7	0.1183
CD4 count (cells/mm^3^), median (IQR)	728 (546-840)	493 (387-572)	268 (249-310)	70 (18-126)	271 (229-328)	P<0.0001
CD8 count (cells/mm^3^), median (IQR)	555 (442-867)	1165 (974-1424)	961 (689-1161)	732 (364-1012)	691 (600-790)	P<0.0001
CD4/CD8 ratio, median (IQR)	1.09 (0.87-1.56)	0.42 (0.29-0.55)	0.28 (0.21-0.39)	0.08 (0.03-0.18)	0.43 (0.36-0.49)	P<0.0001
HIV RNA viral load (log copies/mL), median (IQR)	–	4.16 (3.64-4.85)	4.19 (3.74-4.79)	5.15 (4.63-5.56)	<LDL	P<0.0001

HC, healthy controls; TN, treatment-naive HIV-infected patients; ART, PLWH with treatment over 4 years; M, male; F, female; LDL, lower detection limit. TNs are divided into three subgroups according to blood CD4^+^ T cell count.

*P-value: the difference among HCs, total TNs and ARTs using a Kruskal–Wallis test or Chi-square test.

### Sample collection and processing

The peripheral blood samples were centrifuged (2000rpm/15min) to isolate the plasma. The plasma was then stored at - 80°C for later luminex experiment. PBMCs were collected using standard Ficoll-Paque gradient centrifugation according to the manufacturer’s instructions (Amersham Pharmacia Biotech, Sweden). All samples were processed and analyzed within 24 hours of collection for Flow cytometry and ICS staining.

### Plasma HIV-1 viral load and CD4^+^T-cell count

The plasma HIV-1-RNA levels were measured using a Standard Amplicor HIV Monitor assay, version 1.5 (Roche Diagnostics, Indianapolis, IN, USA), with a lower detection limit of 40 copies/mL. The CD4^+^ T-cell count was determined with a standard flow cytometry technique with a TruCOUNT tube in routinely equipped laboratories (BD Biosciences, San Jose, CA, USA). Both measurements were determined twice per year in a single laboratory using standard methodologies that are included in the National Quality Assurance Programs.

### Immunofluorescence staining and flow cytometric analysis

For surface staining, PBMCs were incubated with directly conjugated antibodies for 30 min at 4°C. Antibodies used included anti-human CD3-BUV737 (clone VCHT1), CD3-BV786 (clone SK7), CD4-BUV395 (clone RPA-T4), CD8-BUV395 (clone RPA-T8), GITR-BV605 (clone V27-580), CD38-BUV737 (clone HB7), CD25-PE-CF594 (clone M-A251, BD Biosciences, San Diego, CA, USA), CD4-APC-fire750 (clone SK3), CD8-BV421 (clone RPA-T8), αβTCR-BV421 (clone IP26), CD56-AF700 (clone 5.1H11), CD56-APC (clone 5.1H11), FasL-PE-Cy7 (clone NDK-1), CTLA-4-BV786 (clone BNI3), CD73-BV711 (clone AD2), HLA-DR-AF700 (clone L243, BioLegend, San Diego, CA, USA), LAG3-APC (clone 3DS223H, Invitrogen, Carlsbad, CA, USA) and the corresponding isotype controls. For intracellular staining of Foxp3 (clone 25901C7, BD Biosciences), Granzyme A-PE-cy7 (clone CB9), granzyme B-APC-fire750 (clone A16A02), perforin-PE-CF594 (clone dG9), Ki-67-BV711 (clone Ki-67, BioLegend) and IDO-APC (clone eyedio, Invitrogen) cells were fixed and permeabilized using Foxp3 Staining Buffer Set (BD Biosciences) according to the manufacturer’s recommendations. A fixable viability dye eFluor 506 (Ebioscience, San Diego, CA, USA) was used to assess cell viability. Data were acquired on a BD LSR Fortessa flow cytometer (BD Biosciences) and analyzed with FlowJo software (Tree Star, Ashland, OR, USA).

### *In vitro* stimulation and intracellular cytokine staining

PBMCs, isolated as described above, were resuspended to 2×10^6^ cells/mL in RPMI 1640 medium plus 10% FBS (R10). Cells were then incubated for 5h at 37°C in a medium with 1 × Cell Stimulation Cocktail (containing 81 nM PMA and 1.34 nM ionomycin plus protein transport inhibitors, Ebioscience). Following incubation, the cells were washed and surface-stained with CD3-BV786 (clone SK7), CD8-BUV395 (clone RPA-T8, BD Biosciences), CD4-APC-fire750 (clone SK3, BioLegend) for 30 minutes in the dark at room temperature, followed by fixation and permeabilization. After permeabilization, cells were stained with IL-10-PE-cy7 (clone JES3-9D7, Ebioscience) antibodies for 50 minutes in the dark at room temperature. A fixable viability dye eFluor 506 (Ebioscience) was used to assess cell viability. Following staining, cells were washed and acquired on an LSRFortessa.

### Quantification of soluble markers

Plasma samples from patients were assayed for the levels of different cytokines and chemokines (G-CSF, GM-CSF, I-TAC, IFN-α, IL1-β, IL-15, IL-6, IL-8, IP-10, MCP-1, MIG and MIP-β) using Luminex multiple kits (Invitrogen, Carlsbad, CA, USA).

### Statistical analysis

The data are expressed as the mean [standard deviation (SD)], median [interquartile range (IQR)], and percentage [frequency]. The normality of each variable was evaluated using the Kolmogorov–Smirnov test. When the data were not normally distributed, the comparison of variables was performed with a Mann–Whitney U-test or a Wilcoxon matched-pairs signed-rank test for unpaired and paired data. A Kruskal–Wallis test followed by Dunn’s multiple comparisons test was applied for comparing two more independent samples. Comparisons of participant characteristics were analyzed using Fisher’s exact test (categorical variables) or the Kruskal–Wallis test (continuous variables). Pearson’s or Spearman correlation coefficients were used to evaluate correlations for normally or non-normally distributed data, respectively. All analyses were performed using GraphPad8 (GraphPad Software, La Jolla, CA, USA). P < 0.05 was considered significant. Heatmaps and the radar map were generated using R version 3.6.3 software (Institute for Statistics and Mathematics, Vienna, Austria; www.r-project.org). 

## Results

### Elevated frequency of Foxp3^+^ DN T cells associated with HIV disease progression

We recruited 121 TNs and gender and age-matched 25 healthy donors. TNs were divided into three groups according to their baseline CD4 levels (< 200 cells/µl, 200-350 cells/µl, and ≥ 350 cells/µl). The demographic and clinical characteristics were illustrated in **Table 1**.

**Figure 1:**
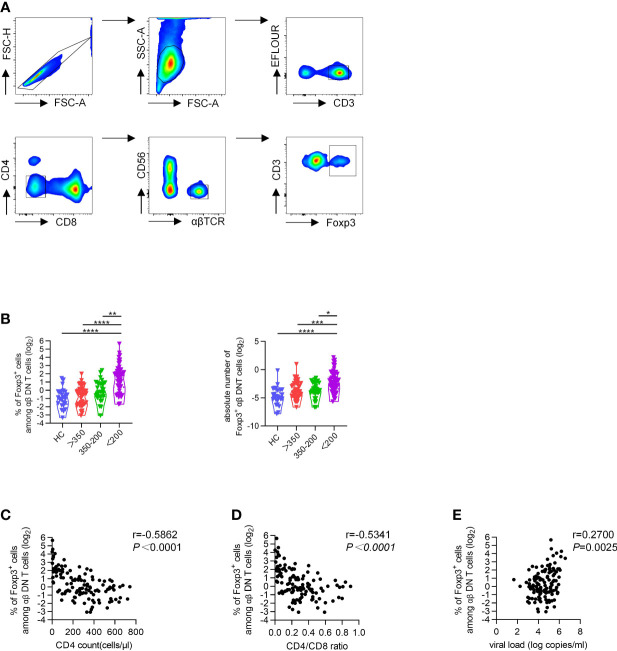
Increased proportions of Foxp3^+^ DNT cells associated with progressive HIV disease. Flow cytometry analysis of Foxp3 expression was performed on PBMCs collected from HCs and different TNs groups. **(A)** Representative flow data showed the expression of Foxp3 on DNT cells from TNs.  **(B)** Violin plots of the percentage of Foxp3^+^ DNT cells from HCs and different TNs groups (n=20-66 each group). P values were obtained by Kruskal-Wallis test followed by Dunn’s multiple comparisons test. **(C–E)** Correlation analysis of the percentages of Foxp3^+^ DNT cells with CD4^+^ T cell count **(C)**, CD4/CD8 ratio **(D)**, HIV viral load **(E)** in PHIV infected patients without treatment. The percentage of Foxp3^+^ DNT cells was represented on a log2 scale. The correlation was calculated using Spearman’s non-parametric test. *P < 0.05, **P < 0.01, ***P < 0.001, ****P < 0.0001.

In line with previous studies, we also found that DNT cells increased with disease progression of HIV infection (**Figure S1A**) (24). To further investigate the potential role of Foxp3 in DNT cells during HIV infection, we performed intracellular flow cytometric analysis of Foxp3 in DNT cells. Representative flow cytometry gating strategy for Foxp3^+^ αβDNT cells was shown in **Figure 1A**. In line with data concluded from a France cohort, we also found comparable frequency and the absolute number of Foxp3^+^ DNT cells among HCs and different groups of PLWH with CD4^+^ T cell count ≥ 200 cells/µl (22). However, PLWH with CD4^+^ T cell count less than 200 cells/µl displayed significantly higher frequency and the absolute number of Foxp3^+^ DNT cells than HCs and patients with CD4^+^ T cell count ≥ 200 cells/µl (**Figure 1B**). Moreover, correlation analysis revealed that the frequency of Foxp3^+^ DNT cells negatively correlated with CD4^+^ T cell count and CD4/CD8 ratio (r = −0.5862, P < 0.0001; r = −0.5341, P < 0.0001, respectively; **Figures 1C, D**). Only a slight positive correlation was observed between the percentage of Foxp3^+^ DNT cells and virus loads (r = 0.27, P = 0.0025; **Figure 1E**). In addition, we also assessed the correlation of DNT cell frequency with CD4^+^ T cell count, CD4/CD8 ratio, and virus load and found no correlation among these parameters (**Figures S1B–D**). Collectively, these results suggested that Foxp3^+^ DNT cells were associated with HIV disease progression, and might be a sign of the severe stage of HIV infection.

### Foxp3^+^ DNT cells correlated with immune activation and systemic inflammation in TNs

**Figure 2:**
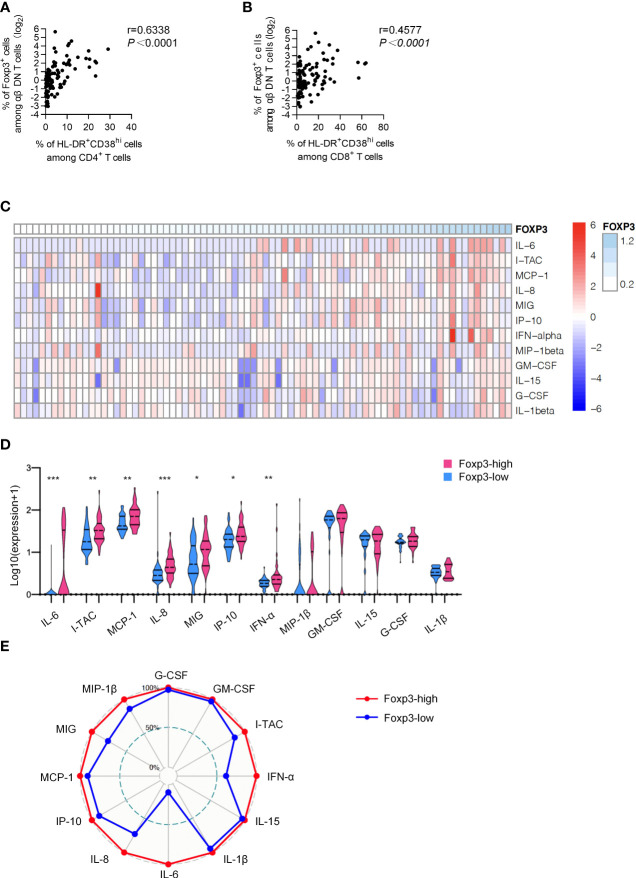
Foxp^3+^ DNT cells correlated with the phenotypic profile of activation and systematic inflammation. **(A, B)** Correlation analysis between the percentage of Foxp3^+^ DNT cells and (HLA-DR^+^CD38^hi^CD4^+^ T cells **(A)** and HLA-DR^+^CD38^hi^CD8^+^ T cells **(B)** from TNs. Spearman’s non-parametric test was used to test for correlations. **(C)** Heatmap depicting the relative concentrations of 12 differentially expressed serum proteins in TNs. Each column of the heatmap indicated a sample, while the rows represented different serum proteins. The color scale in the heatmap represented scores standardized across rows.(blue represented low levels; red, high levels) **(D)** Violin plots of the concentration of twelve differentially expressed serum proteins in the Foxp3-low group (n = 40) and Foxp3-high group (n = 40). The concentration of different serum was shown on a log10 scale. P values were obtained by Kruskal-Wallis test followed by Dunn’s multiple comparisons test. **(E)** Radar map of 12 differentially expressed serum proteins in Foxp3-low group (n = 40) and Foxp3-high group (n = 40). The radius is the percentage of expression. *P < 0.05, **P < 0.01, ***P < 0.001.

To further identify the role of Foxp3^+^ DNT cells in controlling immune activation during HIV infection, we then applied correlation analysis between the frequency of Foxp3^+^ DNT cells and immune activation. It was revealed that the percentage of Foxp3^+^ DNT cells was positively correlated to both CD38^hi^HLA-DR^+^CD4^+^ T cells and CD38^hi^HLA-DR^+^CD8^+^ T cells (r = 0.6338, P < 0.0001; r = 0.4577, P < 0.0001, respectively; **Figures 2A, B**).

Considering a positive feedback loop between pro-inflammatory cytokines and activation of immune cells, we next detected the concentrations of various cytokines and chemokines including G-CSF, GM-CSF, I-TAC, IFN-α, IL1-β, IL-15, IL-6, IL-8, IP-10, MCP-1, MIG and MIP-β in plasma from TNs. As shown in the heatmap, TNs with higher Foxp3 expression on DNT cells displayed higher levels of inflammatory factors in plasma (**Figure 2C**). In addition, we performed a subgroup analysis, dividing TNs into Foxp3-high group (n=40) and Foxp3-low group (n=40) based on the median of Foxp3 expression. The median value 2.26 of Foxp3 expression on DNT cells of the 80 TNs evaluated was used as the cutoff in the present study. Compared to the Foxp3-low group, Foxp3-high patients showed significantly increased levels of inflammatory factors including I-TAC, IFN-α, IL-6, IL-8, IP-10, MCP-1, and MIG (**Figure 2D**). More importantly, levels of I-TAC, IL-6, IL-8, and MCP-1 were positively correlated with the frequency of Foxp3^+^ DNT cells in TNs (**Table 2** and **Figure S2**). Furthermore, we identified overall differences between these two groups using a radar map. Consistently, Foxp3-high patients displayed comprehensively higher levels of pro-inflammatory factors than Foxp3-low patients (**Figure 2E**). Collectively, these data strongly indicated that Foxp3^+^ DNT cells in TNs associated with uncontrolled immune activation and systemic inflammation.

**Table 2:** Correlation Between plasma cytokines and chemokines and the percent of Foxp3^+^ DNT cells in TNs.

Variable	Foxp3	
	r	P-value
G-CSF	0.01753	0.8773
GM-CSF	0.07337	0.5178
**I-TAC**	**0.3848**	**0.0004**
IFN-α	0.2767	0.0130
IL1-β	-0.0001876	0.9987
IL-15	0.03419	0.7633
**IL-6**	**0.3999**	**0.0002**
**IL-8**	**0.3707**	**0.0007**
IP-10	0.2799	0.0119
**MCP-1**	**0.3724**	**0.0007**
MIG	0.2821	0.0112
MIP-β	0.1182	0.2965

P values were obtained by Spearman test (n = 80). Bold fonts indicated strong correlations between the variables and Foxp3.

### Foxp3^+^ DNT cells exhibited a unique characteristic compared with their circulating Foxp3^-^ counterpart in TNs

**Figure 3:**
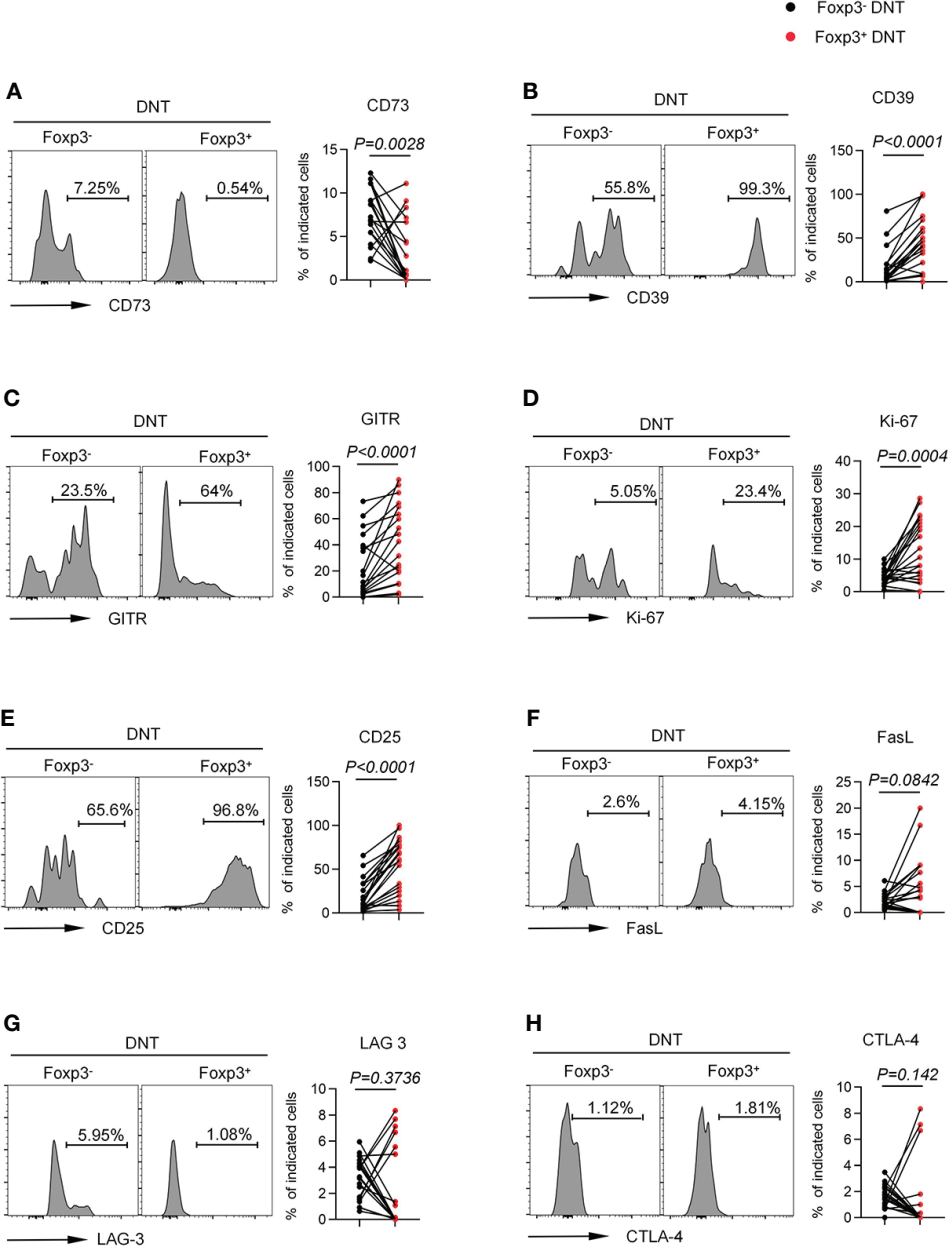
Foxp3^+^ DNT cells showed a unique phenotypic characteristic compared with their circulating Foxp3^-^ counterpart in TNs. Flow cytometry analysis of expression of CD73 **(A)**, CD39 **(B)**, GITR **(C)**, Ki-67 **(D)**, CD25 **(E)**, FasL **(F)**, LAG-3 **(G)** and CTLA-4 **(H)** on Foxp3^-^ *vs*. Foxp3^+^ DNT cells from TNs with baseline CD4^+^ T cell count ≤ 200 cells/µl. Representative histograms (left) and plots (right) displayed the expression of the above markers on Foxp3^-^ *vs*. Foxp3^+^ DNT cells. *P* values were obtained by Wilcoxon matched-pairs signed rank test.

Given that Foxp3 was indispensable to sustaining CD4^+^ Tregs phenotypic stability, metabolic fitness, and regulatory function (27), we wondered the regulatory role of Foxp3 in DNT cells. Accordingly, we compared several membrane-bound or intracellular biomarkers associated with suppressive effects of CD4^+^ Tregs between Foxp3^-^ and Foxp3^+^ DNT cells. Foxp3^+^ DNT cells showed higher expression of CD39 as well as lower expression of CD73 than Foxp3^-^ subset, implying the involvement of ATP/adenosine in Foxp3^+^ DNT-mediated immune suppression (**Figures 3A, B**) (35). Additionally, we found elevated levels of GITR and Ki-67 in Foxp3^+^ DNT cells compared with Foxp3^-^ population, which suggested an enhanced stimulation and proliferation of Foxp3^+^ DNT cells (**Figures 3C, D**) (36). What’s more, Foxp3^+^ DNT cells also preferentially expressed CD25, which was a crucial Treg surface marker implicated in the suppressive function (**Figure 3E**) (27). However, no significant differences were observed in the expression of LAG-3, CTLA-4, and FasL between Foxp3^+^ and Foxp3^-^ DNT cells (**Figures 3F–H**).

**Figure 4:**
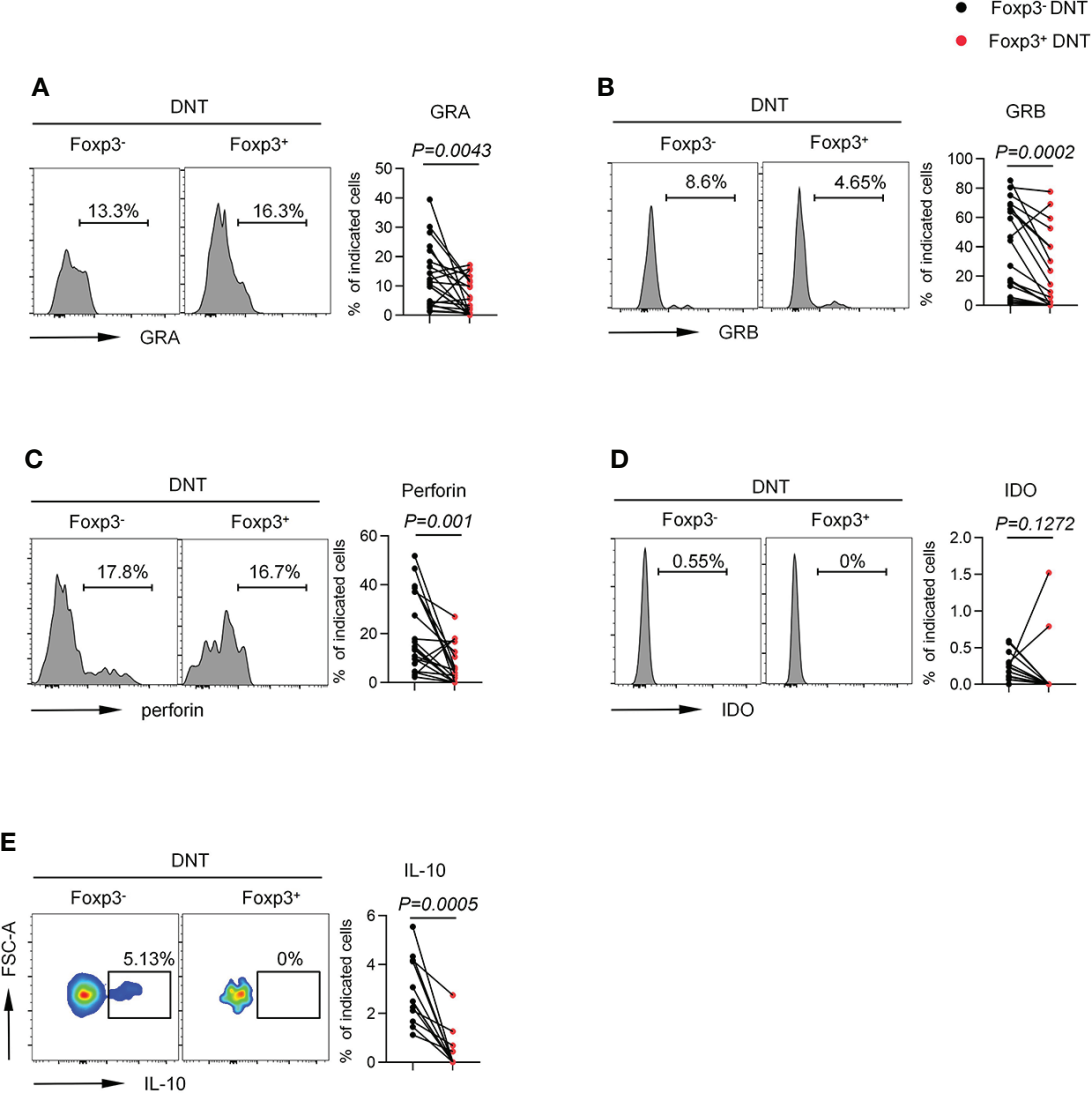
Foxp3^+^ DNT cells secreted different level of cytokine and intracellular proteins compared with their circulating Foxp3^-^ counterpart in TNs. Flow cytometry analysis of expression of GRA **(A)**, GRB **(B)**, Perforin **(C)**, IDO **(D)** and IL-10 **(E)** on Foxp3^-^ *vs*. Foxp3^+^ DNT cells from TNs with baseline CD4^+^ T cell count ≤ 200 cells/µl. Representative histograms (left) and plots (right) displayed the expression of the above markers on Foxp3^-^ *vs*. Foxp3^+^ DNT cells. Statistical tests were performed using the Wilcoxon matched-pairs signed rank test.

To further determine the function of Foxp3^+^ DNT cells, we next detected the ability of DNT cells to secrete cytokines and intracellular proteins. Foxp3^+^ DNT cells produced decreased levels of Granzyme A, Granzyme B and perforin, suggesting an impaired cytolytic capacity (**Figures 4A** **–C**). In addition, Foxp3^+^ DNT cells secreted extremely few levels of IDO and IL-10, two key factors in regulation of immune suppression (**Figures 4D, E**). Taken together, these data indicated a unique phenotypic and intrinsic characteristic of Foxp3^+^ DNT cells, which might exert immune modulatory effects through elevated signaling of CD39, CD25, or vigorous proliferation (high levels of GITR and ki67).

### Increased frequency of Foxp3^+^ DNT cells was partially reversible after ART

**Figure 5:**
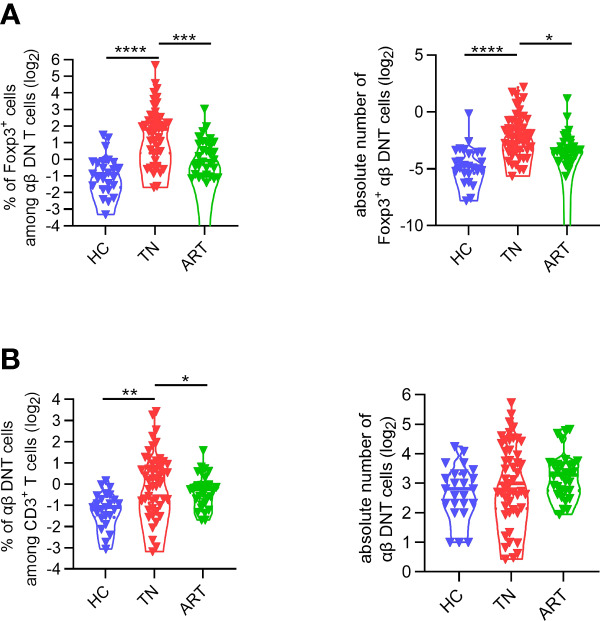
The frequency of Foxp3^+^ DNT cells was partly restored after ART. **(A)**Violin plots of the frequencies (left) and absolute numbers (right) of DNT cells from healthy donors, TNs with baseline CD4^+^ T cell count < 200 cells/µl, ARTs who have been treated more than 4 years with matched nadir CD4^+^ T cell count. **(B)** Comparison of Foxp3^+^ DNT cells frequencies (left) and absolute numbers (right) in healthy donors, TNs, ARTs. *P* values were obtained by the Kruskal–Wallis test, followed by Dunn’s multiple comparisons test. *P < 0.05, **P < 0.01, ***P < 0.001, ****P < 0.0001.

In HIV-infected patients, ART can effectively reduce the overall immune activation and improve CD4^+^ T cell recovery (37). To investigate the impact of ART on Foxp3^+^ DNT cells, we then assessed the expression of Foxp3 on DNT cells among patients experiencing 5.2 years of ART with baseline CD4^+^ T cells count < 200 cells/µl. As shown in **Figure 5A**, the elevated proportion and absolute numbers of Foxp3^+^ DNT cells were significantly decreased in ART-experienced patients compared with that in TN patients. Consistently, the proportion of Foxp3^+^ DNT cells in TNs was downregulated after ART (**Figure 5B**). These data pointed out that Foxp3^+^ DNT cells were partially reversible after ART and mainly played a role in viremic patients, highlighting the significance of Foxp3^+^ DNT cells during HIV infection.

## Discussion

Previous studies have demonstrated that DNT cells accumulated and played a regulatory role in the control of immune activation during HIV infection (20, 22, 24); however, the underlying mechanisms need to be thoroughly explored. In this study, we found a subpopulation of DNT cells which shared the key factor “Foxp3” of CD4^+^ Tregs enriched in PLWH with CD4^+^ T cell count less than 200 cells/µl. Elevated Foxp3^+^ DNT cells were associated with disease progression and immune activation in ART-naïve PLWH. Moreover, we further showed that these Foxp3^+^ DNT cells from PLWH expressed higher levels of CD39, CD25, Ki-67, and GITR, which might play a crucial role in the suppressive regulation mediated by Foxp3^+^ DNT cells. To our knowledge, this is the first time to identify the presence of Foxp3^+^ DNT cells with a unique characteristic in PLWH and its role in HIV disease progression.

However, it is definitely controversial whether DNT cells expressed Foxp3 during HIV infection. In 2012, Petitjean et al. exhibited opposite results to ours, which pointed out that there were few Foxp3^+^ DNT cells in PLWH enrolled in four clinical sites in France (22). The contradiction could be explained by the fact that we used cohorts with distinct characteristics. It is worth noting that we demonstrated the enrichment of Foxp3^+^ DNT cells mainly in PLWH with baseline CD4^+^ T cell count fewer than 200 cells/µl. Meanwhile, Petitjean’s study was based on patients with significantly higher CD4^+^ T cell count (> 334 cells/µl). In fact, these two cohorts were enrolled from two different countries (France and China respectively) with great differences in cultural backgrounds, economic and medical conditions. In Europe, national medical funding accelerated progress in earlier diagnoses and treatment for PLWH, which allowed patients to visit the hospital at the early stage of the infection. However, in most developing counties including China, patients were unaware of their status until the late stage due to the limited medical resource and the lack of propagation of HIV/AIDS science. This led to the consequence that a high percentage of patients were in a state with impaired thymic output and extremely low CD4^+^ T cell count. More importantly, the regulation and maintenance of immune homeostasis might vary depending on different statuses. Our results highlight the importance of Foxp3^+^ DNT in the regulation of immune homeostasis in PLWH, especially in patients with low CD4^+^ T cell count.

Although Foxp3 is considered as a specific marker of CD4^+^ regulatory T cells, it was also reported to express in non-Treg immune cells as well as non-immune cells, including CD8^+^ T cells, γδ T cells, NKT cells, B cells, macrophages, and cancer cells (28–31, 38, 39). Many studies have revealed suppressive effects of Foxp3^+^CD8^+^ Tregs, which showed similar phenotypic features with CD4^+^ Tregs. In untreated PLWH, the proportion of Foxp3^+^CD8^+^ T cells correlated positively with the frequency of HLA-DR^hi^CD4^+^ T cells and inversely with CD4^+^ T cell count, which indicated the involvement of Foxp3^+^CD8^+^ T cells in HIV disease progression (40). As for γδ T cells, Foxp3 was majorly expressed in Vδ1 T cells from tumor-infiltrating lymphocytes (TILs) and contributed to their suppressive function (29). In fact, γδ T cells freshly isolated from peripheral blood barely expressed Foxp3, while Foxp3^+^ γδ T cells could be induced in presence of TGF-β and IL-2/IL-15. The inducible Foxp3^+^ Vδ1 T cells with higher GITR, CTLA-4, and membrane-bound latent form of TGF-β manifested a more potent suppressive effect on responder cells *via* a cell-to-cell contact mechanism compared with Foxp3^-^ Vδ1 T cells. What’s more, the percentage of infiltrating Foxp3^+^CD3^+^CD56^+^ cells in tumors was found to be inversely correlated with patient survival (41). Additionally, lentiviral transduction of Foxp3 into CD3^+^CD56^+^ cells endowed these cells with a potent and cell contact-dependent inhibition on T cell activation. Similarly, apart from being crucial in maintaining food tolerance and tending to decrease RA-associated immunopathology, the high proportion of circulating Foxp3^+^CD19^+^ B cells in MS patients during relapse is a compensatory peripheral response to the inflammatory circumstances of disease activity (38). DC expressing transgene Foxp3 severely limited T-cell proliferation and T cell type-1 immune responses *in vitro (* 31). Collectively, these results suggested the possibility of Foxp3 being expressed in other cells in addition to CD4^+^ Treg cells and involved in the regulation of immune response as a complementary mechanism in the presence of pathological immune imbalance.

It is generally recognized that systemic immune activation is a detrimental consequence of HIV infection. On one hand, it is a crucial driving force of the loss of CD4^+^ T cells, which contributes to the development of AIDS-related events such as opportunistic infections and cancer (3, 4). On the other hand, the enduring immune activation in response to the virus persistence is liable for the growing non-AIDS morbidity and mortality (5, 6). Therefore, it’s imperative to identify an effective intervention targeting HIV-associated immune activation to improve disease management. Physiologically, the immune system has protective mechanisms to avoid the aberrant immune activation that in the case of the disease leads to a marked erosion and offset-tune of the entire immune system. However, CD4^+^ Treg cells, the potent natural regulator which can inhibit T cell activation and proliferation, become draining and dysfunctional during HIV infection, especially in patients with low CD4^+^ T cell count (42). Of note, we found an accumulation of DNT cells, particularly the Foxp3^+^ subset with immune suppressive roles in PLWH. Although DNT cells were also identified as target cells of HIV (43), mature DNT cells were reported to be highly resistant to apoptosis (44). In that condition, due to the incapable CD4^+^ T cells replenishment and impaired thymic output, Foxp3^+^ DNT cells might act as negative feedback to maintain the immune homeostasis.

A key unresolved issue is the specific origin of these Foxp3^+^ DNT cells. Previous studies suggested that high levels of immune activation in PLWH might induce and maintain the population of Tregs as negative feedback (34). Here, we revealed a positive correlation between the frequency of Foxp3^+^ DNT cells and systemic immune activation. Thus, we speculated that these Foxp3^+^ DNT cells proliferated possibly in response to over-activation of the immune system as similar as the expansion of CD4^+^ Tregs in peripheral blood of PLWH, which was confirmed by high expression of Ki-67 in Foxp3^+^ DNT cells. And the elevation of Foxp3^+^ DNT cells might act as negative feedback to ameliorate pathogenesis in infectious diseases. Of note, peripheral DNT cells were reported to derive from CD8^+^ or CD4^+^ T cells in both physiological and pathologic status (10). Due to the serious deficiency of CD4^+^ T cells during HIV infection, it was extremely possible that Foxp3^+^ DNT cells come from CD8^+^ T cells by losing CD8 protein.

An interesting finding in the present study is that Foxp3^+^ DNT cells also expressed CD25, GITR and CD39, which were verified to contribute to immunoregulatory functions of Foxp3^+^CD4^+^ Tregs (27). CD25 rendered Treg cells able to rapidly sense IL-2 produced by self-reactive conventional T cells early in the immune response and compete for growth factors (27). CD39 was an ectoenzyme that converted ATP into adenosine in tandem with CD73, which was reported to involve in the immune regulation mediated by DNT cells in our recent study (24). Additionally, GITR triggering *in vivo* effectively increased the absolute number of cells through enhanced proliferation (36), which was in line with high levels of Ki-67 in Foxp3^+^ DNT cells. In contrast, Foxp3^+^ and Foxp3^-^ DNT cells showed comparable levels of CTLA-4, which prevented costimulation and proliferation of conventional T cells by interacting and downregulating CD80/CD86 on antigen-presenting cells (21). This indicated that the regulatory mechanism of CTLA-4 in DNT cells might be different from that in Tregs, in which foxp3 can form a complex with NFAT to function as an activator of the Ctla4 gene (45). More importantly, both CD4^+^ Tregs and DNT were revealed to exert immune regulation *via* cytolytic activity as well as secretion of IL-10. However, in the present study, Foxp3^+^ DNT cells were found to express lower levels of granzyme A/B and perforin than Foxp3^-^ DNT cells, suggesting the impaired cytolytic capacity of Foxp3^+^ DNT cells. Moreover, these Foxp3^+^ DNT cells even failed to produce IL-10, which was in accordance with limited secretion of IL-10 in Foxp3^+^ CD8^+^ T cells (30). Taken together, the expression of Foxp3 conferred Treg-like features to DNT cells (CD25, GITR, and CD39), while it also accompanied with the loss of some intrinsic functions of DNT cells (granzyme B, perforin and IL-10).

Our study has some limitations. First of all, it is difficult to confirm the suppressive role of Foxp3^+^ DNT cells by performing functional *in vitro* experiments, due to lack of methods to isolate viable Foxp3^+^ cells. Second, according to the fact that PLWH with low CD4^+^ T cell count are more susceptible to opportunistic infection, we could not distinguish the effects of HIV itself or opportunistic pathogens responsible for our findings. Finally, a heavily gender-skewed cohort was applied in the present study, which was almost completely made up of men who have sex with men (MSM). It is well acknowledged that women were in a higher immune activation and more inflammatory state during HIV infection (46). Hence, we need to verify our findings in a gender-balanced cohort.

In summary, our study demonstrated that elevated Foxp3^+^ DNT cells with Treg-like phenotype were associated with systemic immune activation and disease progression during HIV infection, providing a promising clinical intervention for the control of immune activation.

## Data availability statement

The original contributions presented in the study are included in the article/**Supplementary material**. Further inquiries can be directed to the corresponding authors.

## Ethics statement

The studies involving human participants were reviewed and approved by the Committee of Ethics at Beijing Ditan Hospital. The patients/participants provided their written informed consent to participate in this study.

## Author contributions

LZ performed the experiments, analyzed the data and wrote the manuscript. YW and DW performed the experiments and analyzed the data. XW, BL, MJ, MZ, NC, MD, CS, DC, JX, LW and HL collected samples, and performed the experiments. HZ participated in the critical review of the manuscript and revised the manuscript. YK designed the experiments, analyzed the data and revised the manuscript. All authors contributed to the article and approved the submitted version.

## Funding

This work was supported by Beijing Municipal Natural Science Foundation for Distinguished Young Scholars (JQ21023), National Natural Science Foundation of China (82171548, 81971307), Beijing Municipal Administration of Hospitals’ Ascent Plan (DFL20191802), and Beijing Municipal Administration of Hospitals Clinical Medicine Development of Special Funding Support (ZYLX202126).

## Acknowledgments

The authors sincerely thank all the patients and healthy donors in this study.

## Conflict of interest

The authors declare that the research was conducted in the absence of any commercial or financial relationships that could be construed as a potential conflict of interest.

## Publisher’s note

All claims expressed in this article are solely those of the authors and do not necessarily represent those of their affiliated organizations, or those of the publisher, the editors and the reviewers. Any product that may be evaluated in this article, or claim that may be made by its manufacturer, is not guaranteed or endorsed by the publisher.
