# Supplementary material for: Elevated Foxp3+ double-negative T cells are associated with disease progression during HIV infection
Source: Front Immunol. 2022 Jul 28;13:947647. doi: 10.3389/fimmu.2022.947647 (PMC9365964; doi:10.3389/fimmu.2022.947647)
Supplement: Supplementary file 1 [file DataSheet_1.pdf]

Supplementary Material

Supplementary Figures

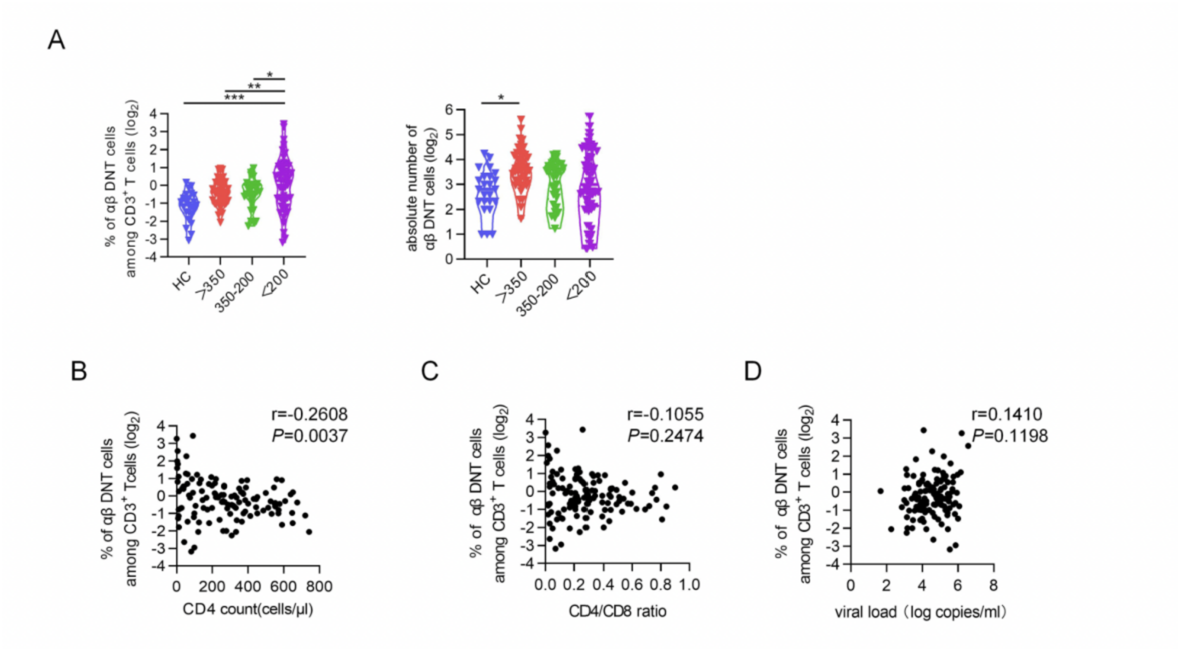

Fig. S1 Detection of absolute number and frequencies of DNT cells in TNs.

(A)The proportions (left) and absolute numbers (right) of DNT cells in healthy donors and different PLWH groups ($CD4^+$ T cells count ⩾ 350 cells/μl, 200 – 350 cells/μl, < 200 cells/μl). *P* values were obtained by the Kruskal–Wallis test, followed by Dunn's multiple comparisons test. (B-D) Correlation analysis of the percentages DNT cells with $CD4^+$ T cell count (B), CD4/CD8 ratio (C), and HIV viral load (D) in untreated HIV-infected individuals. The percentage of Foxp3+ DNT cells was represented on a log2 scale. $*P < 0.05$, $**P < 0.01$,$***P < 0.001$.

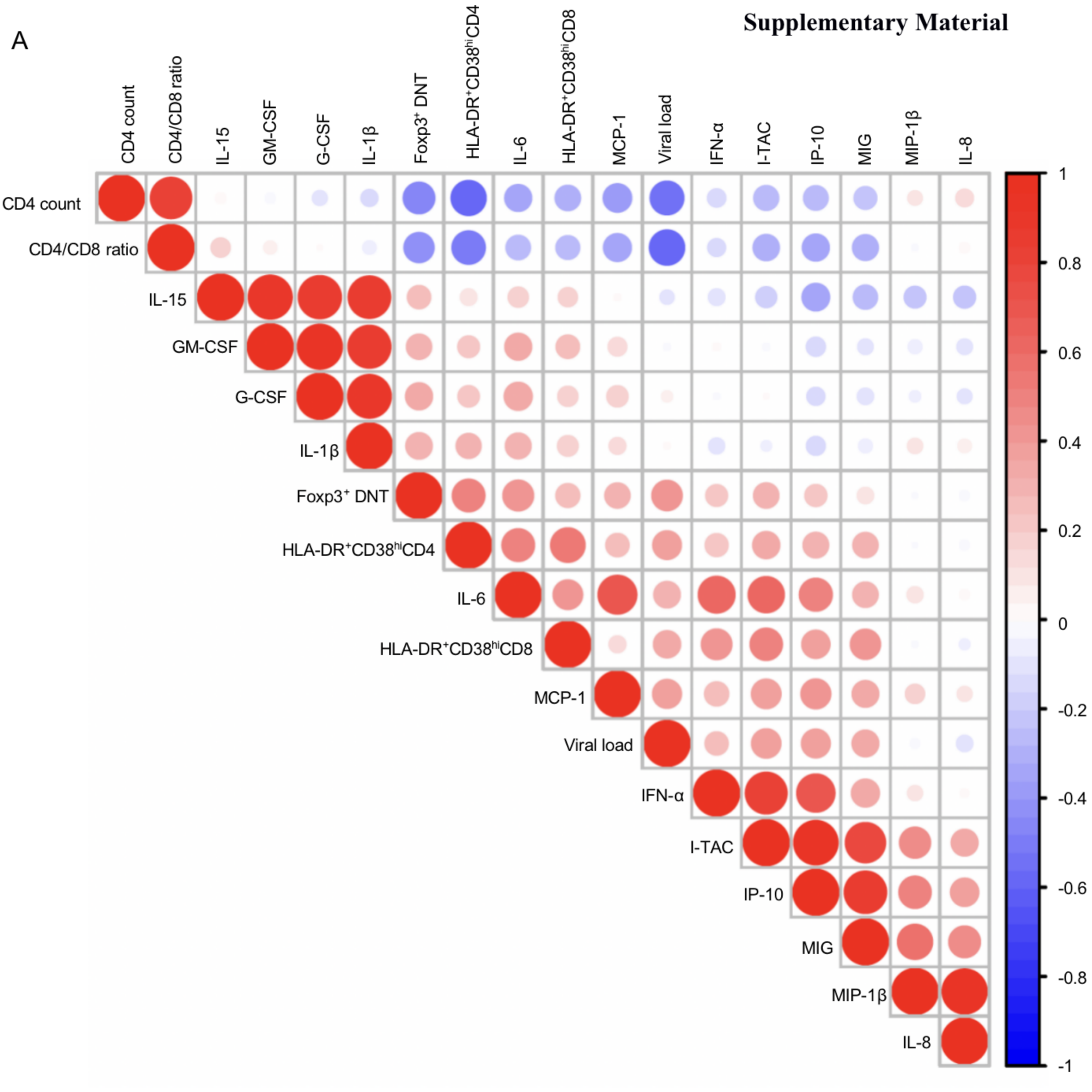

Fig.S2 Correlation of frequencies of Foxp3^{+} DNT cells with inflammation and clinical indicators in TNs.

(A)Dot heatmap of the correlation between selected proteins and clinical indicators. The size and color density of circles were proportional to the correlation between 2 variables in TNs.
